# Gender-Specific Predictors of Depressive Symptoms among Community Elderly 

**Published:** 2017-04-29

**Authors:** Mahbobeh Faramarzi, Mahla Cheraghi, Mohammad Zamani, Farzan Kheirkhah, Ali Bijani, Seyed Reza Hosseini

**Affiliations:** ^1^ Social Determinants of Health (SDH) Research Center, Babol University of Medical Sciences, Babol, Iran; ^2^ Student Research Committee, Babol University of Medical Sciences, Babol, Iran; ^3^ Cancer Research Center, Babol University of Medical Sciences, Babol, Iran; ^4^ Department of Psychiatry, Babol University of Medical Sciences, Babol, Iran

**Keywords:** Cognitive Dysfunction, Sex, Depressive Disorder, Comorbidity

## Abstract

**Background:** We aimed to determine the gender-specific predictors of depressive symptoms among an Iranian elderly community population.

**Study design:** A cross-sectional study.

**Methods:** This study was performed on elderly subjects (aged ≥60 yr) who participated in the Amirkola Health and Aging Project, Amirkola, Babol, northern Iran in 2011-12. Depression was assessed by the Geriatric Depression Scale. Fourteen variables, including marital status, age, education, occupation, living alone, social support, dependency in daily activities, physical activity, smoking, body mass index, chronic pain, medicine use, comorbidities, and cognitive impairment, were analyzed as predictors of depression.

**Results:** In males, age group of 80-84 yr (odds ratio (OR)=0.22, 95% confidence interval (CI): 0.09, 0.55), occupation (OR=0.62, 95% CI: 0.42, 0.90) and social support (OR=0.82, 95% CI: 0.77, 0.88) had protective effects against depression, and smoking (OR=1.67, 95% CI: 1.15, 2.44), cognitive impairment (OR=2.18, 95% CI: 1.34, 3.45) and comorbidities(OR=1.42, 95% CI: 1.27, 1.60) were found as risk factors. In females, social support (OR=0.97, 95% CI: 0.65, 1.44) and higher education (OR=0.10, 95% CI: 0.01, 0.84) were two protective factors against depression, and being unmarried (OR=1.88, 95% CI: 1.13, 2.35), cognitive impairment (OR=1.50, 95% CI: 1.01, 2.21), comorbidities(OR=1.30, 95% CI: 1.18, 1.44) and chronic pain (OR=1.57, 95% CI: 1.01, 2.44) were four positive predictors of depression.

**Conclusions:** There were both similarities and differences in predictors of depression between old males and females. These findings suggest physicians and healthcare executives consider genderspecific risk/protective factors to improve preventive mental health programs in older males and females

## Introduction

The aging population in the world is growing. Nine hundred one million people aged 60 yr and older were estimated in the world population in 2015, and this number is predicted to reach 1.4 billion by 2030. The growth of the older population is projected to be rapid in Asia, with an increase of more than 60% between 2015 and 2030, compared to a 23% increase in Europe^1^. Iran’s rate of elderly population increase was estimated to be 5.2% in 2011^2^. Depression is seen frequently in elder people, with prevalence rates ranging from 25% to almost 50%^3^, and it usually appears as comorbid disorders, leading to reduction in life quality and increase in mortality rate^4^. The prevalence of depression in the Iranian elderly population was 36.7% in 2012^5^. Majdi et al. reported some psychosocial factors including living alone, source of income, and supporting system were predictors of depression in Iranian elderly population. There was no relationship between depression of the population and gender and education level^6^.

Studies on the risk factors of depression in the elderly have shown that many variables can potentially be associated with this disease including low body mass index (BMI)^7^, chronic diseases^8^, physical decline^9^, low socioeconomic status^10^, low family support^11^, cognitive impairment^12^, marriage status^13^, pain^14^, and metabolic syndrome^15^. Regarding the association between depression and gender, little information is available needs more clarification. While female gender is considered one of the most important risk factors for depression in some studies^16^, others do not confirm this view^17, 18^.

The current study is the first survey assessing the relationship between gender and depressive symptoms among Iranian elderly community population in Babol, northern Iran. Thus, we tested the hypothesis whether there are similarities and differences in predictors of depression between old males and females. The identification of gender-specific predictors of depressive symptoms should prove useful for developing programs that are more effective on prevention and intervention in elderly population.

## Methods

### Study population

This cross-sectional study was conducted on the elderly who participated in the “Amirkola Health and Aging Project” (AHAP) ^19^ from April 2011 to July 2012. It is a cohort study evaluating the medical and psychological health and life quality of elderly individuals living in Amirkola, Babol. The total number of registered elderly population was 1,616 ^19^.

### Data collection

The health center staff filled out demographic questionnaires consisting of three category items regarding the demographic factors (including participants’ age, sex, marital status, education, and job), habits (smoking), and self-perceived health (chronic pain, use of medicines, and comorbidity of disease). All participants were included the study except who filled the questionnaires incompletely. Comorbidity of disease was ascertained by asking if the subjects were diagnosed by physicians, or currently were receiving treatment for any of the following diseases: heart disease; stroke; hypertension; diabetes; respiratory disease; gastrointestinal disease; osteoporosis; arthritis; thyroid disease; trauma; mental illness; visual/hearing impairment; cancer etc.

### Measurements

**Geriatric Depression Scale (GDS):** Depression symptoms in the elderly were examined using the GDS. It contains 15 questions and each question is scored 0 or 1^5, 20, 21^. Based on the scores, the subjects were divided into four groups: normal (0–4); mild depression (4–8); moderate depression (9–11), and severe depression (12–15)^22^. GDS is a validated questionnaire in the Iranian population^23^. Cronbach’s alpha for GDS was reported 0.81 in this study.

**Activities of Daily Living Scale (ADL) and Instrumental Activities of Daily Living Scale (IADL):** ADL and IADL are two instruments that screen elderly respondents for independence in daily activities. ADL contains seven items of activities of daily living, including feeding, bathing, grooming, dressing, bowel control, bladder control, toileting, and ambulation. The score range for each item is from 0 to 2. A higher score reflects a better degree of independence in activities of daily living^24^. The IADL scale contains eight items of activities instrumental for daily living including: the ability to use a telephone; shopping; food preparation; housekeeping; laundry; mode of transportation; responsibility for own medications; and ability to handle finance. The score ranges from 0 to 2^25^. The content validity of the Persian ADL & IADL was 0.82^26^.

**Mini Mental State Examination (MMSE):** The MMSE is the most commonly used instrument to evaluate cognitive status in the elderly. It contains 11 items and evaluates five cognitive functions, including orientation, attention, memory, language and visual-spatial skills. The maximum score is 30 points and a score of <23 reflect cognitive impairment ^27^.

**Physical Activity Scale for the Elderly (PASE):** Physical activity was evaluated by the PASE, designed for the self-assessment of physical activity over a one-week period. The PASE assesses walking, light/moderate or strenuous sports activities, strength training, activities in the household or leisure-time activities, and voluntary work. The scale is scored from 0 to 400 or up. The PASE has been validated and has acceptable test-retest reliability ^28^.

**Duke Social Support Index (DSSI):** Social support was assessed using the shortened version of the DSSI. It consists of 11 questions and uses a Likert-type scale and is scored as: 1 (rarely/very dissatisfied); 2 (sometimes/dissatisfied); and 3 (most of the times/satisfied). Total DSSI scores ranged from 11 to 33. A higher score of DSSI indicated higher levels of social support. Internal consistency and test-retest reliability were high ^29^. We used the validated Persian version of DSSI^30^. Cronbach’s alpha of DSSI was 0.69 in this study.

### Statistical analysis

Descriptive analyzes were conducted to compute the percentages, means, and standard deviations of variables. Chi-square and Student’s *t*-test were used for assessing the association of depression with qualitative and quantitative variables, respectively. To determine the final predictors of depression in females and males, the following factors were included in the two series of logistic regression analysis as independent variables: marital status; age; education; occupation; living alone; social support; dependency in daily activity; physical activity; smoking; BMI; chronic pain; number of comorbidities; and cognitive impairment. Significance levels were set at *P*<0.05 and all tests were two-tailed.

### Ethical issues

This study was approved by the Ethics Committee of Babol University of Medical Sciences and Health Services. Informed consent was obtained from all subjects.

## Results

Overall, 210 subjects were excluded. Of the 1,396 participants, 46% were female, 57.9% were less than 70 yr, and 7.1% were educated 12 years or more (Table 1).

**Table 1:** Demographic characteristics of the participants

**Variables**	**Number**	**Percent**
Gender		
Male	753	54.0
Female	643	46.0
Age (yr)		
60-64	520	37.3
65-69	288	20.6
70-74	246	17.6
75-79	216	15.5
80-84	84	6.0
<85	42	3.0
Marital status		
Married	1183	84.7
Single/divorced/ widowed	213	15.3
Educational level		
Literacy	886	63.5
Primary school	411	29.4
High school/University	99	7.1
Occupational status		
Employed	446	31.9
Unemployed	950	62.5

Tables 2 and 3 show the relationship between the depressive symptoms and the independent variables based on Chi-square and *t*-test analyzes, respectively. The prevalence of depressive symptoms was 42.6% (95% CI: 40.0, 45.2), which was significantly higher in females (59.3%, 95% CI: 55.4, 63.1) than in males (28.4%, 95% CI: 25.2, 31.6). As for the total sample, being unmarried, living alone, unemployment, chronic pain, disability in daily activity, cognitive impairment, higher BMI, comorbidities, low social support and medicine use were significantly associated with depressive symptoms. Regarding gender, unmarried status, cognitive impairment, comorbidities, low social support and medicine use were significantly correlated with depression in both males and females. In addition, living alone, no job, smoking and lower BMI were related to depressive symptoms in males, but not in females. On the other hand, lower education, chronic pain and higher age were associated with depression in females only.

**Table 2:** Relation between qualitative variables and depression in males and females

	**Male (n=753**)			**Female (n=643**)			**Total (n=1396**)		
	**Depression+** **n=214**	**Depression-** **n=539**		**Depression+** **n=381**	**Depression-** **n=262**		**Depression+** **n=595**	**Depression-** **n=801**	
**Variables**	**n (%)**	**n (%)**	***P*** ** value**	**n (%)**	**n (%)**	***P*** ** value**	**n (%)**	**n (%)**	***P*** ** value**
Marital status			0.001			0.001			0.001
Married	191 (23.7)	509 (72.7)		267 (55.3)	216 (44.7)		458 (37.7)	725 (61.3)	
Single/divorced/widowed	23 (43.4)	30 (56.6)		114 (71.3)	46 (28.8)		137 (64.3)	76 (35.7)	
Living alone			0.017			0.296			0.001
Yes	12 (50.0)	12 (50.0)		48 (74.9)	26 (35.1)		60 (61.2)	38 (38.8)	
No	535 (41.2)	763 (58.8)		333 (58.5)	236 (58.5)		535 (41.2)	763 (58.8)	
Education			0.281			0.001			0.069
Literacy	135 (29.9)	316 (70.1)		278 (63)	161 (37.0)		409 (46.2)	477 (53.8)	
Primary school	60 (28.0)	154 (72.0)		106 (53.8)	91 (46.2)		166 (40.4)	245 (59.6)	
High school/university	19 (21.6)	69 (78.4)		1 (9.1)	10 (90.1)		20 (20.2)	79 (79.8)	
Job			0.001			0.315			0.001
Yes	102 (23.7)	329 (65.6)		7 (46.7)	8 (53.3)		109 (24.4)	337 (75.6)	
No	86 (22.6)	295 (77.4)		374 (59.6)	254 (40.4)		486 (51.2)	464 (48.8)	
Chronic Pain			0.315			0.001			0.001
Yes	128 (34.4)	244 (65.6)		321 (63.2)	187 (36.8)		449 (51.0)	431 (49.0)	
No	86 (22.6)	254 (77.4)		60 (44.4)	75 (55.6)		146 (28.3)	370 (71.7)	
Smoking			0.001			0.343			0.117
Yes	98 (37.5)	163 (62.5)		4 (80.0)	1 (20.0)		102 (38.3)	164 (61.7)	
No	116 (23.6)	376 (76.4)		377 (59.1)	261 (40.9)		493 (43.6)	637 (56.4)	
Dependent to daily activity			0.541			0.108			0.035
Yes	5 (35.7)	9 (64.3)		16 (76.2)	5 (23.8)		21 (60.0)	14 (40.0)	
No	209 (28.3)	530 (71.7)		365(58.7)	257(41.3)		574 (42.2)	787 (57.8)	

**Table 3:** Relationship beween quantitative varibales and depression in males and females

**Variables**	**Male (n=753**)			**Female (n=643**)			**Total (n=1396**)		
	**Depression+** **n=214**	**Depression-** **n=539**	***P*** ** value**	**Depression+** **n=381**	**Depression** **n=262**	***P*** ** value**	**Depression+** **n=595**	**Depression-** **n=801**	***P*** ** value**
	**Mean (SD)**	**Mean (SD)**		**Mean (SD)**	**Mean (SD)**		**Mean (SD)**	**Mean (SD)**	
Age (yr)	70.1 (7.3)	69.5 (7.6)	0.350	68.7 (7.0)	67.6 (6.6)	0.040	69.2 (7.1)	68.9 (7.4)	0.390
Cognitive impairment	25.7 (3)	26.7 (2.7)	0.001	23.7 (3.9)	24.8 (3.8)	0.001	24.4 (3.2)	26.1 (3.7)	0.001
Body mass index	25.7 (4.1)	26.1 (4.1)	0.200	28.5 (4.7)	28.5 (4.8)	0.860	27.5 (4.7)	26.9 (4.4)	0.020
Number of comorbidities	2.7 (1.9)	1.8 (1.4)	0.001	3.9 (2)	2.8 (1.7)	0.001	3.5 (2)	2.1 (1.6)	0.001
Physical activity	99.9 (69.4)	103.2 (65.2)	0.530	110 (54.2)	121 (54.1)	0.001	106.4 (60.3)	109.2 (62.3)	0.390
Social support	27.3 (3.2)	28.8 (2.5)	0.001	26.4 (3.3)	27.5 (3.3)	0.001	26.7 (3.3)	28.4 (2.8)	0.001
Number of medicines	2.6 (2.8)	1.7 (2.2)	0.001	3.9 (2.7)	3 (2.5)	0.001	3.4 (2.3)	2.2 (2.4)	0.001

Table 4 presents the relationship between predictors of depression in both genders based on the logistic regression model. For males, age range of 80–84 years (OR=0.22, 95% CI: 0.09, 0.55) and 85–99 yr (OR=0.31, 95% CI: 0.11, 0.87), being employed (OR=0.61, 95% CI: 0.42, 0.90), and social support (OR=0.82, 95% CI: 0.77, 0.88) had protective effects against depressive symptoms, whereas smoking (OR=1.67, 95% CI: 1.15, 2.44), cognitive impairment (OR=2.18, 95% CI: 1.34, 3.45) and comorbidities (OR=1.42, 95% CI: 1.27, 1.60), were risk factors.

**Table 4:** Relationship between indipendent variables and depression in males and females

**Variables**	**Male (n=753)** **OR (95% CI)**	***P*** **-value**	**Female (n=643)** **OR (95% CI)**	***P*** ** value**
Marital status				
Married	1.00		1.00	
Single/divorced/widowed	1.69 (0.68, 4.16)	0.252	1.88 (1.13, 2.35)	0.015
Living alone				
No	1.00		1.00	
Yes	1.21 (0.34, 4.28)	0.764	0.828 (0.42, 1.60)	0.577
Education				
Literacy	1.00		1.00	
Primary school	1.45 (0.92, 2.28)	0.106	0.84 (0.56, 1.26)	0.409
High school/university	1.24 (0.62, 246)	0.538	0.10 (0.01, 0.84)	0.039
Age (yr)				
60-64	1.00		1.00	
65-69	0.79 (0.45, 1.29)	0.318	0.98 (0.57, 1.69)	0.967
70-74	1.26 (0.74, 2.15)	0.377	2.27 (1.08, 4.75)	0.029
75-79	0.72 (0.40, 1.29)	0.272	1.38 (0.53, 3.57)	0.497
80-84	0.22 (0.09, 0.55)	0.001	1.82 (0.53, 6.21)	0.337
85-99	0.31 (0.11, 0.87)	0.027	1.27 (0.18, 8.79)	0.807
Occupational status				
Employed	1.00		No data	
Unemployed	0.61 (0.42, 0.90)	0.014	No data	
Having chronic pain				
No	1.00		1.00	
Yes	1.29 (0.89, 1.86)	0.173	1.57 (1.01, 2.44)	0.041
Smoking status				
Nonsmoker	1.00		No data	
Smoker	1.67 (1.15, 2.44)	0.007	No data	
Dependent to daily activity				
No	1.00		1.00	
Yes	0.49 (0.13, 1.74)	0.271	1.29 (0.42, 3.96)	0.656
Cognitive impairment				
No	1.00		1.00	
Yes	2.18 (1.34, 3.54)	0.002	1.50 (1.01, 2.21)	0.040
Body mass index (kg/m^2^)				
<25	1.00		1.00	
25-29.9	1.36 (0.88, 2.11)	0.156	0.95 (0.59, 1.52)	0.839
>30	1.12 (0.61, 2.05)	0.698	0.80 (0.48, 1.33)	0.395
Comorbidity				
No	1.00		1.00	
Yes	1.42 (1.27, 1.60)	0.001	1.30 (1.18, 1.44)	0.001
Physical activity				
No	1.00		1.00	
Yes	0.76 (0.47, 1.25)	0.291	0.97 (0.65, 1.46)	0.656
Social support				
No	1.00		1.00	
Yes	0.82 (0.77, 0.88)	0.001	0.92 (0.86, 0.97)	0.001

For females, two factors had protective effect against depressive symptoms and included social support (OR=0.92, 95% CI: 0.65, 1.44) and higher education (OR=0.10, 95% CI: 0.01, 0.84), while four factors were positive predictors, and included unmarried status (OR=1.88, 95% CI: 1.13, 2.35), cognitive impairment (OR=1.50, 95% CI: 1.01, 2.21), comorbidities (OR=1.30, 95% CI: 1.18, 1.44) and chronic pain (OR=1.57, 95% CI: 1.01, 2.44).

Four common factors, which were living alone, dependency in daily activity, BMI, and physical activity, were not predictors of depressive symptoms in both genders.

## Discussion

Our results showed that predictors of depression had similarities and differences in males and females. Cognitive impairment and comorbidities were two common positive predictors of depressive symptoms in both genders, and social support was the only similar negative predictor. Four common factors, which were living alone, dependency in daily activity, BMI, and physical activity, were not associated with depression in both genders.

We found that the prevalence of depressive symptoms was more common in females than males. This finding is in accordance with another study reporting an association of gender with elderly depression^31^. Many different explanations for gender differences in depression have been proposed. Biological descriptions for females’ greater vulnerability to depression focused on the direct effects of ovarian hormones on females’ moods, or moderating effects of hormones (adrenal hormones) on responses to stress. In addition, low social power of females contributes to their vulnerability to major traumas more so than males. Traumas may directly (making them helpless to control their lives) or indirectly (increasing their reactivity to stress) result in depression ^32^.

In this research, unmarried status was the most powerful positive predictor of elderly depression in females. Lupa et al. in a population-based sample of age 75 yr and older, reported that divorced or widowed marital status was significantly correlated with depression ^31^.

In males, as mentioned above, higher range of ages was a protective factor against depression, whereas in females, increasing age was positively associated with depressive symptoms. There are controversial results about the relationship between age and depression. In a study, the negative associations between depressive symptoms and age were reported^33^. In contrast, depression increased in the elderly aged >75 yr rather than in the elderly <75 yr. One of the explanations for the effect of age on prognosis of depression in old people is probably related to factors, such as previous episodes and medical comorbidities ^34^.

In this study, chronic pain predicted depression in females. It is difficult to specify whether elderly depression causes chronic pain or vice versa. In a co-twin control study, the relationship between depression symptoms and low back pain was evaluated after adjustment for genetics. In addition to the explanation of the correlation of depression with back pain, the authors stated this relationship was stronger in the dizygotic case-control analysis compared with the analysis of monozygotic case-control ^14^.

We found that higher education (12 yr or more) in females was a negative predictor of depression. Lower education is a risk factor for elderly depression ^31^. Females with little education usually cannot receive a prompt diagnosis and treatment when suffering diseases. It seems that poor health and low mental status make them more prone to depressive symptoms.

Cognitive impairment increased the risk of depression in both male and female elderly subjects. This finding is in accordance with previous studies reporting a positive association between cognitive impairment and depression ^12, 31^. It is not easy to determine whether depression causes cognitive impairment or vice versa. Depression might cause cognitive impairment, while cognitive impairment may results in depression ^35^.

We found that social support was a preventive factor against depression in both males and females. Faramarzi et al. similarly reported that social support was a negative predictor of depression in the elderly ^16^. People with more social support are better protected from the negative consequences of psychological symptoms of illness, and their quality of life will be enhanced.

We observed that being employed had a negative association with depression in the male elderly. A study reported occupational and social activities reduced the chance of suffering from depression ^5^.

Our findings showed that living alone, dependent daily activity, BMI, and physical activity were not associated with depressive symptoms in males and females, whereas another study was in contradiction with our results ^21^.

How can we explain the differences in the predictors of depressive symptoms between males and females? Elderly depression results directly in more stressful experiences by interfering with occupational and social support, and in predisposition to stress by stealing the individual of any sense of mastery, and probably sensitizing the biological systems involved in the stress response. As mentioned above, lack of social power in females leads them to be more disposed to depression compared with males. Additionally, most of the females are under working pressure everyday due to their social status and roles more than males (such as childcare, domestic work, jobs, and caring for sick and elderly family members). Some elderly females also have more difficulty in making money than males and are more likely to live in poverty, especially in developing countries. In the present study, social support scores were higher in males than females. Females usually suffer from stressors more than males and are more prone to poor health. This can be explained in that females biologically and psychologically are more hyper-responsive to stress than males ^32^.

This study had a few limitations. The cross-sectional nature of the study does not considerably help to find causal relationships. Prospective cohort studies are a more reliable way of determining the gender-specific predictors of depressive symptoms. Besides, to get more detailed and comprehensive views on the depression, further investigations are needed using substitute procedures, for example clinical diagnosis. Another limitation might be that data on illness-related information are missing such as the number of previous depressive episodes and number of hospitalization. In addition, more researches are suggested to define the associations between predictors of depression and social culture. In addition, it is recommended that diagnostic criteria for major depressive episode (DSM) be used instead of GDS in future studies.

Further surveys are suggested to assess the relationship between cultural variables and gender-specific predictors of depression. Although this study had some weak points, it also had strong points, including the large sample size, the high response rate, the use of validated scales to measure depressive symptoms, and assessing large numbers of risk factors (14 independent variables).

## Conclusions

There were both similarities and differences in predictors of depression between males and females. Future studies should also include longitudinal follow-ups to explain further the differences in the predictors of depressive symptoms between females and males. It is proposed that physicians and healthcare professionals consider gender-specific risk/protective factors to improve preventive mental health programs in older males and females.

## Ethical considerations

All aspects of the publication ethics, including plagiarism, data fabrication and/or falsification, double publication and/or submission, informed consent, redundancy, etc. have been observed by the authors.

## Acknowledgements

The authors would like to thank the Deputy for Research of Babol University of Medical Sciences for the financial support and to Dr. Evangeline Foronda for the English editing.

## Conflict of interest statement

Authors have declared that no competing interests exist.

## Funding

This paper is retrieved from a doctoral thesis. The Deputy Research of Babol University of Medical Sciences supported the funding (Grant Number: 1868).

## Highlights

- About half of the subjects had depression (twice as common in females as in males).
- In males, occupation and social support were protective factors against depression.
- Cognitive impairment and comorbidities were risk factors for depression in males.
- Social support and higher education protected females against depression.
- Being unmarried and chronic pain were positive predictors of depression in females.
